# Performance and validation of a digital memory test across the Alzheimer’s disease continuum

**DOI:** 10.1093/braincomms/fcaf024

**Published:** 2025-01-17

**Authors:** Sofia Toniolo, Bahaaeddin Attaallah, Maria Raquel Maio, Younes Adam Tabi, Elitsa Slavkova, Verena Svenja Klar, Youssuf Saleh, Mohamad Imran Idris, Vicky Turner, Christoph Preul, Annie Srowig, Christopher Butler, Sian Thompson, Sanjay G Manohar, Kathrin Finke, Masud Husain

**Affiliations:** Nuffield Department of Clinical Neurosciences, University of Oxford, Oxford OX3 9DU, UK; Cognitive Disorders Clinic, JR Hospital, Oxford OX3 9DU, UK; Nuffield Department of Clinical Neurosciences, University of Oxford, Oxford OX3 9DU, UK; Centre for Preventive Neurology, Queen Mary University of London, London E1 4NS, UK; Nuffield Department of Clinical Neurosciences, University of Oxford, Oxford OX3 9DU, UK; Nuffield Department of Clinical Neurosciences, University of Oxford, Oxford OX3 9DU, UK; Nuffield Department of Clinical Neurosciences, University of Oxford, Oxford OX3 9DU, UK; Department of Experimental Psychology, University of Oxford, Oxford OX2 6GG, UK; Nuffield Department of Clinical Neurosciences, University of Oxford, Oxford OX3 9DU, UK; Cognitive Disorders Clinic, JR Hospital, Oxford OX3 9DU, UK; Nuffield Department of Clinical Neurosciences, University of Oxford, Oxford OX3 9DU, UK; Nuffield Department of Clinical Neurosciences, University of Oxford, Oxford OX3 9DU, UK; Department of Neurology, Memory Center, Jena University Hospital, Jena 07747, Germany; Department of Neurology, Memory Center, Jena University Hospital, Jena 07747, Germany; Cognitive Disorders Clinic, JR Hospital, Oxford OX3 9DU, UK; Department of Neurology, Imperial College London, London W12 0NN, UK; Cognitive Disorders Clinic, JR Hospital, Oxford OX3 9DU, UK; Nuffield Department of Clinical Neurosciences, University of Oxford, Oxford OX3 9DU, UK; Cognitive Disorders Clinic, JR Hospital, Oxford OX3 9DU, UK; Department of Experimental Psychology, University of Oxford, Oxford OX2 6GG, UK; Department of Neurology, Memory Center, Jena University Hospital, Jena 07747, Germany; Department of Psychology, Ludwig-Maximilians-University Munich, Munich 80802, Germany; Nuffield Department of Clinical Neurosciences, University of Oxford, Oxford OX3 9DU, UK; Cognitive Disorders Clinic, JR Hospital, Oxford OX3 9DU, UK; Department of Experimental Psychology, University of Oxford, Oxford OX2 6GG, UK

**Keywords:** Alzheimer’s disease, online testing, cognition, dementia, digital biomarkers

## Abstract

Digital cognitive testing using online platforms has emerged as a potentially transformative tool in clinical neuroscience. In theory, it could provide a powerful means of screening for and tracking cognitive performance in people at risk of developing conditions such as Alzheimer’s disease. Here we investigate whether digital metrics derived from an in-person administered, tablet-based short-term memory task—the ‘What was where?’ Oxford Memory Task—were able to clinically stratify patients at different points within the Alzheimer’s disease continuum and to track disease progression over time. Performance of these metrics compared to traditional neuropsychological pen-and-paper screening tests of cognition was also analysed. A total of 325 people participated in this study: 49 patients with subjective cognitive decline, 57 with mild cognitive impairment, 63 with Alzheimer’s disease dementia and 156 elderly healthy controls. Most digital metrics were able to discriminate between healthy controls and patients with mild cognitive impairment and between mild cognitive impairment and Alzheimer’s disease patients. Some, including Absolute Localization Error, also differed significantly between patients with subjective cognitive decline and mild cognitive impairment. Identification accuracy was the best predictor of hippocampal atrophy, performing as well as standard screening neuropsychological tests. A linear support vector model combining digital metrics achieved high accuracy and performed at par with standard testing in discriminating between elderly healthy controls and subjective cognitive decline (area under the curve 0.82) and between subjective cognitive decline and mild cognitive impairment (area under the curve 0.92), while performing worse in classifying between mild cognitive impairment and Alzheimer’s disease patients (area under the curve 0.75). Memory imprecision was able to predict cognitive decline on standard cognitive tests over one year. Overall, these findings show how it might be possible to use a digital memory test in clinics and clinical trial contexts to stratify and track performance across the Alzheimer’s disease continuum.

## Teaser

Toniolo *et al*. report that performance at a tablet-based short-term memory task, the ‘What was where?’ The Oxford Memory Task, was able to clinically stratify patients at different stages of Alzheimer’s Disease, track disease progression longitudinally, predict cognitive decline on standard cognitive tests after 1 year and correlate to hippocampal atrophy.

## Graphical Abstract

**Graphical abstract:**
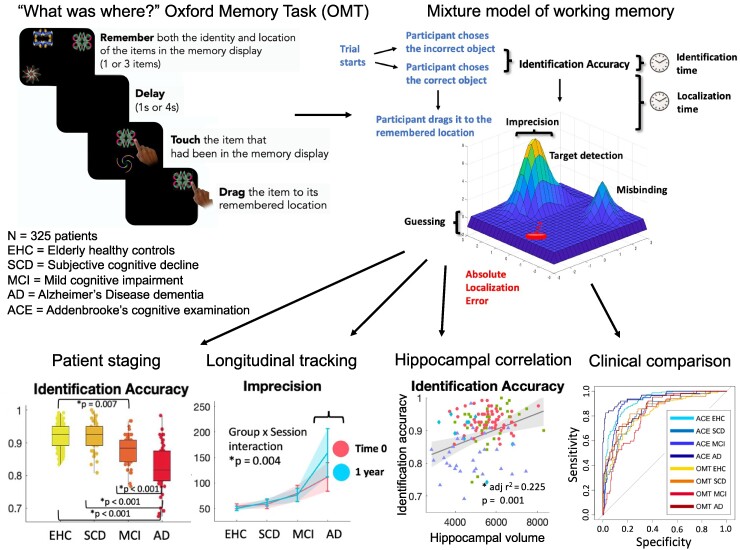

## Introduction

Digital cognitive testing is being increasingly deployed as screening tool for patients at risk of developing Alzheimer’s disease, for recruitment in clinical trials and longitudinal follow-up performed remotely.^1,2^ Some tests can detect subtle signs of cognitive impairment that cannot be captured by standard clinical assessments.^3^ This makes their deployment potentially extremely valuable for large-scale screening purposes in early phases of the disease when cognitive impairment is at subthreshold levels on current scales.^4^ Visual short-term memory (STM) tests have been extensively deployed in patients at risk of developing Alzheimer’s disease using digital platforms.^4,5^ Two types of paradigms have been used. The first involves change-detection tasks, in which participants indicate whether a change occurred or not in visual displays of differing numbers of items in a binary correct/incorrect fashion.^6^ The second uses delayed-reproduction tasks, where participants are asked to reproduce features of the remembered item (e.g. its location, colour or orientation) in a continuous response space. This allows modelling of the responses according to a resource model,^7^ where quantity (number of items held in memory) can be traded for quality (precision of recall of each item). The more items stored, the lower their precision in memory.

Mixture modelling approach is a very influential computational model of how visual STM resources are allocated.^8^ In this model, each response can be classified according to four different factors: probability of correctly identifying a target (**target detection**); erroneously placing an object at the location of another item in memory (**misbinding**); **random guessing** about the features of an item and **precision of memory** (probability distribution of the responses around the target).^8^ Previous analysis of performance on a delayed reproduction task, the ‘What was where?’ Oxford Memory Task (OMT), demonstrated that people with a genetic risk factor for familial Alzheimer’s disease (FAD), such as carriers of the Presenilin-1 mutation, exhibit higher misbinding rates, and that their recall correlated with the degree of hippocampal atrophy.^4^ Recent evidence using the same paradigm showed increased misbinding also in patients with sporadic, late-onset Alzheimer’s disease.^9,10^ Whether this relates to hippocampal integrity is still unknown. Increased misbinding seems to be consistent across many different pathologies that target the hippocampus, including autoimmune limbic encephalitis,^11^ surgical resection due to epilepsy surgery,^12^ infectious encephalitis^13^ and anoxia.^14^ However, these are all relatively rare conditions, and extensive data on more common neurodegenerative conditions such as late-onset Alzheimer’s disease is missing.

Another important gap in the literature is the limited choice of digital outcomes used. Most previous studies have focused solely on misbinding rates, which is only one of a wide array of metrics that can be computed using digital delayed-reproduction tasks. This is important as there is evidence for selective disease-specific impairment in some cognitive metrics, whilst others are spared (e.g. increased misbinding but not guessing in patients with hippocampal pathology and higher rates of guessing but not misbinding in patients with Parkinson’s Disease).^15^ Further, while we know that the degree of hippocampal atrophy relates to standard screening tests of cognitive function,^16^ and that delayed-reproduction visual STM metrics also show a good concordance with measures of hippocampal integrity,^4^ there is surprisingly little published data on head-to-head comparisons between digital STM metrics and commonly used clinical cognitive scales in predicting hippocampal integrity.

Longitudinal data on the temporal evolution of digital metrics, including misbinding, in patients at risk of developing Alzheimer’s disease are also surprisingly scarce. At present, to our knowledge, only one study has reported on performance over time in a cohort of people with familial Alzheimer’s disease, both symptomatic (*n* = 6) and presymptomatic (*n* = 23) gene mutation carriers.^5^ The authors used the ‘What was where?’ OMT and found that identification accuracy declined over time only in symptomatic carriers. Moreover, localization error was greater the closer the presymptomatic individuals were to their estimated year of onset of dementia. Crucially, a standard delayed memory task was only able to detect a significant difference between this group and controls one year later compared with localization error performance on the digital test. Despite these encouraging findings in familial Alzheimer’s disease, the performance of late-onset Alzheimer’s disease patients on this digital task has not been extensively characterized.

Here we report findings in a large group of individuals across the Alzheimer’s disease continuum, including people with subjective cognitive decline (SCD), mild cognitive impairment (MCI) and established clinical Alzheimer’s disease dementia. In this study, we sought to establish whether deficits on digital metrics can be detected before a clinical diagnosis of Alzheimer’s disease dementia and if these might also help to discriminate between clinical groups and elderly healthy controls (EHC). We tested individuals cross-sectionally and performed longitudinal assessment in a subset. Further, we investigated which among the digital metrics was the best predictor of cognitive decline longitudinally. The relationship of digital metrics to hippocampal integrity was also examined. Finally, a linear support vector machine^17^ was used to test the utility of digital metrics in classifying participants, and the resulting model was subsequently compared with one using a standard cognitive screening test.

## Materials and methods

### Participants

A total of 325 participants were enrolled in the study: 49 people with SCD, 57 with MCI, 63 patients with Alzheimer’s disease dementia and 156 EHC. Patients were recruited from cognitive disorders clinics at the John Radcliffe Hospital in Oxford, United Kingdom and Friedrich-Schiller-Universität Klinik, Jena, Germany. Recruitment and data collection occurred between 2017 and 2021. SCD was defined according to the 2020 criteria from Jessen *et al.* for subjective cognitive decline.^18^ MCI patients were classified according to Petersen’s criteria of 2014.^19^ Alzheimer’s disease dementia patients were defined as having Alzheimer’s disease clinical syndrome according to the 2018 criteria by Jack *et al.*^20^ and will be subsequently referred to as Alzheimer’s disease. From the Oxford cohort, 26 of the total 63 Alzheimer’s disease dementia patients had plasma biomarker analysis for Alzheimer’s disease biomarkers, with the results corroborating their diagnosis based on clinical assessment, neuropsychological testing, MRI and fluorodeoxyglucose positron emission tomography. All subjects underwent either brain computed tomography or MRI imaging and were excluded from the study if there was evidence of structural abnormalities not compatible with their clinical diagnosis. Elderly healthy subjects who reported any psychiatric or neurological illness or were on psychoactive drugs were excluded from the study. All participants had normal or corrected-to-normal vision acuity and no colour blindness. A summary of participants’ demographics is presented in Table 1.

**Table 1:** Demographics and tests

	EHC	SCD	MCI	AD	*P*-value all	*P*-value EHC/SCD	*P*-value SCD/MCI	*P*-value MCI/AD
Age	67.3 (8.6)	57.6 (7.8)	69.7 (8.9)	70.6 (9.0)	* < 0.001	* < 0.001	* < 0.001	0.582
Gender (M/F)	58/98	23/26	39/18	34/29	* < 0.001	0.222	*0.025	0.105
Education	14.9 (4.1)	15.3 (4.7)	11.8 (3.2)	13.2 (3.7)	* < 0.001	0.480	* < 0.001	0.136
Handedness (R/L/A)	128/26/2	45/4/0	49/6/2	57/3/3	0.150	0.305	0.799	0.470
ACE	95.9 (3.1)	92.7 (6.3)	87.9 (3.8)	73.9 (15.7)	* < 0.001	*0.013	*0.004	* < 0.001
DS	17.6 (4.4)	17.4 (5.2)	14.8 (3.3)	14.2 (4.5)	* < 0.001	1.0	*0.008	1.0
HADS	8.3 (5.6)	23.0 (3.7)	11.5 (6.8)	9.2 (6.5)	* < 0.001	* < 0.001	* < 0.001	0.129
GDS	2.4 (2.7)	6.2 (3.8)	5.1 (3.8)	3.8 (3.6)	* < 0.001	* < 0.001	0.097	0.066
PSQI	5.6 (3.1)	9.3 (4.4)	6.3 (3.6)	5.1 (3.6)	* < 0.001	* < 0.001	* < 0.001	0.163

* Denotes statistically significant results.

ACE, Addenbrooke’s Cognitive Examination-III; DS, Digit Span; HADS, Hospital Anxiety and Depression Scale; GDS, 15-item Geriatric Depression Scale; PSQI, Pittsburgh Sleep Quality Index; M, male; F, female; Education is reported in years; R, right-handed; L, left-handed; A, ambidextrous.

A subset of 60 people from the Oxford cohort took part in the longitudinal part of the study and completed the repeated assessment at 1 year. Of these 60 participants, there were 21 EHC, 15 SCD, 12 MCI, and 12 were patients with Alzheimer’s disease dementia. A summary of participants’ demographics is presented in Supplementary Table 1. The smaller numbers for the longitudinal dataset are due to the study being prematurely interrupted due to COVID-19 restrictions. While we have subsequently developed a fully remote, online version of this task,^21^ the data reported here are from a tablet version that required face-to-face administration.

One hundred thirty eight participants from the Oxford cohort (EHC: *n* = 61, SCD, *n* = 31, MCI, *n* = 9, Alzheimer’s disease *n* = 37) agreed to a 3T structural MRI scan. Demographics and standard tests of cognition for this subsample are presented in Supplementary Table 2.

The study was performed in accordance with the ethical standards as laid down in the 1964 Declaration of Helsinki and its later amendments. Ethical approval was granted by the University of Oxford ethics committee (IRAS ID: 248379, Ethics Approval Reference: 18/SC/0448) and the local ethics committee in Jena. All participants gave written informed consent prior to the start of the study.

### Neuropsychological and behavioural test assessment

#### Cross-sectional assessment

The study protocol included a brief neuropsychological assessment and the ‘What was where?’ OMT.^4,9,11^ The neuropsychological assessment included measures of global cognition, verbal STM, depression, and sleep quality to rule out a potential neurodegenerative disorder or major depression in the elderly controls and to be able to compare performances at standard cognitive tests to our experimental paradigm. These included Addenbrooke's cognitive examination (ACE-III, subsequently termed ACE),^22^ Digit Span (DS),^23^ Hospital Anxiety and Depression Scale (HADS),^24^ 15-item Geriatric Depression Scale (GDS)^25^ and Pittsburgh Sleep Quality Index (PSQI).^26^ Participants’ test scores are presented in Table 1. Tests were performed in English for participants living in the UK and in German for those living in Germany.

**Figure 1:**
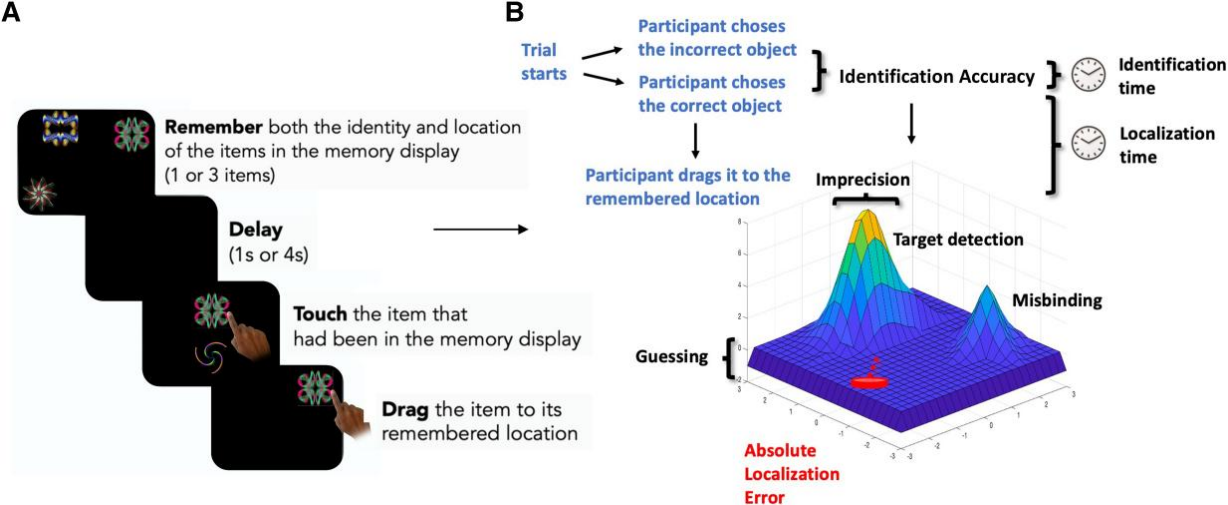
**‘What was where?’ OMT.** (**A**) This panel illustrates the task design. Participants were presented with either 1 or 3 fractals randomly distributed on the screen. After a 1 or 4 s delay two fractals appeared at the centre of the screen, one of which had appeared in the memory array whereas the other one was a distractor. Firstly, they needed to identify the object they had seen previously (‘what’), and then drag it back to its original location (‘where’). (**B**) The task provides four basic performance metrics: Identification accuracy; Absolute Localization Error; Identification time and Localization time. In addition, using the Mixture Model, it is possible to assess levels of Target detection, Misbinding, Guessing and Imprecision.

A schematic of the ‘What was where?’ OMT is shown in Fig. 1A. Stimuli were presented on a black background and were chosen from a library of 196 fractals (http://sprott.physics.wisc.edu/fractals.htm), containing 49 different shapes of 4 colour variations each. Participants sat ∼30 cm in front of a tablet (either iPad or Android), yielding 2.3° of visual angle. Stimuli were calibrated using the dimension on the screen to ensure matching of stimuli properties across different tablet models. A fully remote online version of this task, available for computers, laptops, tablets and phones, has been subsequently developed (https://oxfordcognition.org/) and was used in subsequent studies.^21^

In different trials, participants were presented with either 1 or 3 fractals located randomly on the screen. They were asked to remember the identity of the fractals (‘what’), and their locations (‘where’). After a delay of either 1 or 4 s, two fractals appeared at the centre of the screen along the vertical axis. One of these had appeared in the memory array (target), whereas the other one was a foil, which had not been shown in the current trial. Participants were required to touch the target and drag it to its original location. They were instructed to be as precise as possible, thus allowed to move the item and adjust its location as many times as they needed. This design yielded four different conditions (1 and 3 items, 1 and 4 s). Each participant performed a practice block of 8 trials followed by three test blocks of 40 trials, with a total of 120 test trials (10 trials × 4 conditions × 3 blocks). The order of the trials in a block was randomly chosen for each block. The locations of the fractals were determined by a MATLAB script (MathWorks, Inc.) in a pseudorandom manner. Written instructions were provided in English for all participants as embedded in the task, while oral instructions were given in German for participants tested in Germany and in English for participants tested in the UK.

Overall, this task allowed us to extract different working memory metrics (see Fig. 1B), including (**basic metrics**):

- **Identification accuracy**: the proportion of trials in which participants correctly identified the target.
- **Absolute localization error**: the Euclidian distance from the centre of original item location to the centre of participant’s response location.
- **Identification time**: the time in seconds taken to identify the correct object.
- **Localization time**: the time in seconds to drag the chosen object to its remembered location.

The Mixture Model of working memory by Bays *et al.*^14^ was then fitted to the data to unravel the differential contribution of memory errors to our dataset. Model fitting was achieved using a permutation approach, where for each single trial, we calculated the distances between the response location and the location of:

1. Target.
2. Distractor (non-target in that trial).
3. A distractor taken from a randomly chosen trial.

Depending on which of these distances was the shortest, the response was either counted as target (1), distractor (2, i.e. misbinding) or random response (3, i.e. uniform guessing). We repeated this procedure 5000 times per trial, introducing a distractor from a randomly chosen trial each time. This procedure allowed us to calculate proportions for these three sources of response per trial (absolute amount of response type/5000). The introduction of a distractor that was randomly chosen from another trial allowed us to differentiate whether an error was systematically linked to the very specific trial’s distractor or whether it could be accounted for even by a randomly chosen distractor that was not present at trial. Importantly, these metrics were calculated uniquely on trials where an object was correctly identified. This approach has been previously published using this task and subsequent variations.^9,27^

The following metrics (**mixture model metrics**) were therefore calculated:

- **Target detection:** the probability of correctly identifying the target.
- **Misbinding:** the probability of mislocalizing a correctly identified item to the remembered location of another item in the memory array.
- **Guessing:** the probability of random guessing responses.
- **Imprecision:** the width of the distribution of the responses around the target.

#### Longitudinal assessment

At both visits all subjects completed the ‘What was where?’ OMT, the ACE-III and DS. The summary of demographics and test scores can be found in Supplementary Table 1. Group differences in demographics and test scores were calculated using the same principles of the cross-sectional assessment.

### MRI acquisition and analysis

T1-weighted volumetric MR brain images were acquired on a 3T Siemens Magnetom Verio syngo scanner using a magnetization prepared rapid gradient echo protocol acquired in sagittal orientation (TR = 2000 ms, TE = 1.94 ms, TI = 880 ms, flip angle = 8 degrees, FOV read = 256 mm and voxel size = 1.0 × 1.0 × 1.0 mm). All images were reviewed by a trained neurologist to exclude the presence of remarkable macroscopic brain abnormalities not compatible with the original diagnosis. Hippocampal volumes (HV) were estimated using FSL-FIRST.^28^ For each participant, left and right HV were calculated, and bilateral HV was computed. We also calculated whole brain volumes for each subject. We subsequently computed the head size-corrected values for whole brain volumes and HV, using the scaling factor derived from SIENAX.^29^ When referring to HV and whole brain volumes throughout the article, only head size-corrected volumes have been used. Images were carefully visually inspected after each processing step.

### Statistical analysis

All analyses were conducted using MATLAB 2019a, R (version 3.5.2) and JASP (JASP team, 2022). Data visualization was conducted using software Grammar of graphics plotting in MATLAB R2018b (Gramm library)^30^ and RStudio (Version 1.1.463). Statistical significance was set as *P* < 0.05, two-tailed.

#### Cross-sectional analysis

A one-way ANOVA was used to compare subjects’ continuous variables in demographics and test scores, with Holm *post hoc* correction amongst the four groups, while χ^2^ test was used to compare categorical variables between groups. Additionally, a cumulative measure for each metric was derived by calculating the mean across the four conditions (1 item 1 s, 1 item 4 s, 3 items 1 s, 3 items 4 s). An ANCOVA, with age, gender, and education as covariates, with subsequent Holm *post hoc* correction, was used to test differences across the groups on each cumulative metric while controlling for factors that differed across groups and could impact performance.

We also examined the effects of Set size and Delay across all groups (transdiagnostically), calculating a 2 (set size: 1 item, 3 items)×2 (delay: 1 s, 4 s) ANOVA for each of the digital working memory metrics. Effect size was quantified using Eta Squared (*η*^2^), defined as large (*η*^2^ > 0.14), medium (*η*^2^ > 0.06) or small (*η*^2^ > 0.01).^31^

#### Longitudinal analysis

A 4 (Group)×2 (Session) ANCOVA, with age, gender and education as covariates, with subsequent Holm *post hoc* correction, was used to test differences across the groups and sessions on each cumulative metric. Additionally, we tested whether the baseline values extracted from our tests could be used to predict cognitive decline over one year at standard neuropsychological tests. To this end, we calculated the change between ACE scores from the second visit and the first visit and looked at whether any of the metrics was able to predict cognitive decline after 1 year. We ran linear regression for each metric separately and compared the results with the Cocor package in RStudio.^32^

#### MRI analysis

A generalized linear model was used to study correlations between neuropsychological measures and HV, while correcting for age, gender and education. Regression coefficients were compared using the Cocor package in RStudio.^32^

#### Linear support vector machine

A linear support vector machine classifier (Classification learner, Matlab) was used to test the performance of a model including age, gender, education and the eight digital metrics derived from the ‘What was where?’ OMT, in discriminating between the four different diagnostic groups (EHC, SCD, MCI and Alzheimer’s disease), and a competing model using age, gender, education, and ACE scores, also derived from the same dataset.

## Results

### Demographics and standard neuropsychological tests

Age, gender, and education were not matched across groups, and therefore were included as covariates when comparing group performances (Table 1). As expected, ACE scores were statistically significantly different across groups, with Alzheimer’s disease patients showing the lowest scores, followed by MCI, then SCD and finally EHC. DS scores were not different between EHC and SCD but declined significantly in MCI and Alzheimer’s disease patients. Patients with SCD scored higher on questionnaires of depression and quality of sleep compared with the other groups.

### Cross-sectional analysis

**Figure 2:**
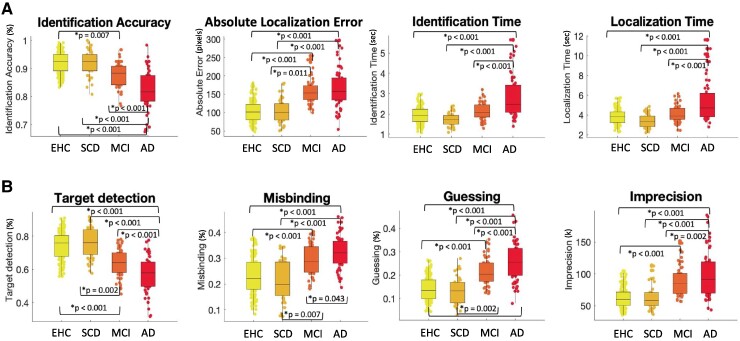
**Cross-sectional analysis.** (**A**) Between-group analysis for basic performance metrics. (**B**) Between-group analysis for mixture model metrics. An ANCOVA, with age, gender, and education as covariates, with subsequent Holm *post hoc* correction, was used to test differences across the groups. Participants are represented as unique datapoints.

All digital metrics were able to discriminate between the groups, with a high effect size for a between-group difference (Table 2, column ALL, Fig. 2). They were able to discriminate between EHC and Alzheimer’s disease and SCD and Alzheimer’s disease (Table 2, columns EHC/AD and SCD/AD). No metric was able to distinguish between EHC and SCD, which highlights the fact that despite these patients’ complaints, they perform within normal range on the visual STM test used here. On the other hand, compared with healthy controls, patients with MCI showed lower Identification Accuracy, had higher Absolute Localization Error rates, lower rates of Targets detected, higher amount of Guessing, higher rates of Misbinding, and higher memory Imprecision.

**Table 2:** Summary results from the between-group analysis

	ALL	EHC/SCD	EHC/MCI	SCD/MCI	EHC/AD	SCD/AD	MCI/AD
Identification accuracy	*F* = 38.2, *P < 0.001 *η*^2^ = 0.251	*t* = 1.8, *P* = 0.139, *d* = 0.322	*t* = 3.0, *P = 0.007,*d* = 0.504	*t* = 0.8, *P* = 0.403, *d* = 0.182	*t* = 10.7, *P < 0.001, *d* = 1.646	*t* = 6.3, *P < 0.001, *d* = 1.324	*t* = 6.2, *P < 0.001, *d* = 1.141
Absolute localization error	*F* = 27.0, *P < 0.001 *η*^2^ = 0.184	*t* = −1.5, *P* = 0.124, *d* = −0.273	*t* = −5.4, *P < 0.001, *d* = −0.909	*t* = −2.9, *P = 0.011, *d* = −0.6	*t* = −8.5, *P < 0.001, *d* = −1.314	*t* = −4.9, *P < 0.001, *d* = −1.041	*t* = −2.2, *P* = 0.058, *d* = −0.405
Identification time	*F* = 27.9, *P < 0.001 *η*^2^ = 0.205	*t* = 0.6, *P* = 0.506, *d* = 0.118	*t* = −2.3, *P* = 0.073, *d* = −0.374	*t* = −2.3, *P* = 0.073, *d* = −0.492	*t* = −8.8, *P < 0.001, *d* = −1.358	*t* = −5.3, *P < 0.001, *d* = −1.476	*t* = −5.3, *P < 0.001, *d* = −0.984
Localization time	*F* = 27.4, *P < 0.001 *η*^2^ = 0.199	*t* = 0.8, *P* = 0.423, *d* = 0.142	*t* = −2.2, *P* = 0.059, *d* = −0.369	*t* = −2.8, *P* = 0.067, *d* = −0.502	*t* = −8.7, *P < 0.001, *d* = −1.342	*t* = −7.0, *P < 0.001, *d* = −1.484	*t* = −5.3, *P < 0.001, *d* = −0.972
Target detection	*F* = 37.5, *P < 0.001 *η*^2^ = 0.246	*t* = 0.9, *P* = 0.361, *d* = 0.162	*t* = 5.4, *P < 0.001, *d* = 0.900	*t* = 3.4, *P = 0.002, *d* = 0.738	*t* = 10.3, *P < 0.001, *d* = 1.587	*t* = 6.7, *P < 0.001, *d* = 1.426	*t* = 3.7, *P < 0.001, *d* = 0.687
Misbinding	*F* = 20.5, *P < 0.001 *η*^2^ = 0.154	*t* = −0.3, *P* = 0.764, *d* = −0.053	*t* = −4.3, *P < 0.001, *d* = −0.720	*t* = −3.1, *P = 0.007, *d* = −0.667	*t* = −7.4, *P < 0.001, *d* = −1.147	*t* = −5.2, *P < 0.001, *d* = −1.094	*t* = −2.3, *P = 0.043, *d* = −0.427
Guessing	*F* = 42.0, *P < 0.001 *η*^2^ = 0.268	*t* = −1.2, *P* = 0.245, *d* = −0.206	*t* = −5.6, *P < 0.001, *d* = −0.920	*t* = −3.3, *P = 0.002, *d* = −0.714	*t* = −11.0, *P < 0.001, *d* = −1.688	*t* = −7.0, *P < 0.001, *d* = −0.768	*t* = −4.2, *P < 0.001, *d* = −0.768
Imprecision	*F* = 24.7, *P < 0.001 *η*^2^ = 0.179	*t* = −1.4, *P* = 0.141, *d* = −0.261	*t* = −4.1, *P < 0.001, *d* = −0.672	*t* = −1.9, *P* = 0.120, *d* = −0.411	*t* = −8.5, *P < 0.001, *d* = −1.311	*t* = −5.0, *P < 0.001, *d* = −1.050	*t* = −3.4, *P = 0.002, *d* = −0.639

Statistically significant values are represented in bold.

Absolute Localization Error, Target detection, Misbinding and Guessing were also able to discriminate between people with SCD and MCI (Fig. 2, Table 2, column SCD/MCI). However, the precision of their memory recall (Imprecision), as well as the proportion of correctly identified items (Identification Accuracy) were not different between those groups. Absolute Localization Error was the only metric that was not able to track disease progression between patients with MCI and Alzheimer’s disease dementia, probably because it was already quite high in patients with MCI (Fig. 2A, Table 2, column MCI/AD). In the comparison between MCI and Alzheimer’s disease dementia, Misbinding was significantly higher in patients with Alzheimer’s disease dementia, but in this sample the metric had the lowest of all effect sizes (Fig. 2B, Table 2, column MCI/AD).

There was a main effect of Set size, of large magnitude (*η*^2^ > 0.14) in all metrics, except Imprecision, where it was of medium magnitude (*η*^2^ > 0.06), (Supplementary Tables 3 and 4, Supplementary Figs 1 and 2). Set size for Misbinding was not included because it cannot be computed for 1 item, as at least two objects are required for misbinding to occur. All metrics, except for Misbinding, also showed an effect of Delay, which however was of small (*η*^2^ > 0.01) to medium (*η*^2^ > 0.06) magnitude. Identification time, Identification accuracy, Absolute Localization Error and memory Imprecision also showed a significant Set Size by Delay interaction, which was however of small magnitude (*η*^2^ > 0.01), with a synergistic impact of higher number of items and longer delays determining worse memory performance.

### Longitudinal analysis

#### Group and session effects

**Figure 3:**
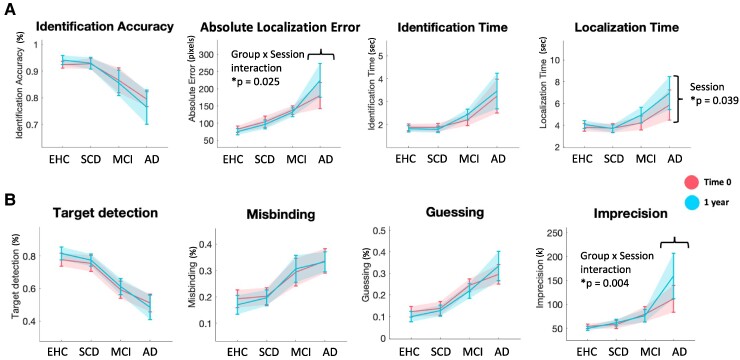
**Longitudinal analysis.** (**A**) Longitudinal analysis of basic performance metrics. (**B**) Longitudinal analysis of mixture model metrics. Baseline session (Time 0) in coral red, follow-up session after 1 year (1 year) in light blue. A 4 (Group)×2 (Session) ANCOVA, with age, gender and education as covariates, with subsequent Holm *post hoc* correction was used to test differences across groups and sessions.

All metrics showed a significant effect of Group, with a high effect size (*η*^2^ > 0.14), even if the sample size was much smaller compared with the bigger cross-sectional dataset (Fig. 3, Supplementary Table 5). Only Localization Time showed a main effect of Session (*F* = 4.3, *P = 0.039, *η*^2^ = 0.024) (Fig. 3, Supplementary Table 5). Absolute Localization Error and memory Imprecision showed a significant Group by Session interaction (Fig. 3, Supplementary Table 5). *Post hoc* analysis for Absolute Localization Error and Imprecision showed a significant difference between sessions only in the Alzheimer’s disease group (Absolute Localization Error: *t* = −3.120, *P = 0.031, *d* = −1.274, Imprecision: *t* = −4.064, *P = 0.002, *d* = −1.660).

As a comparison, we also computed the effects of Group and Session for the two neuropsychological tests used, the ACE and DS (Supplementary Fig. 3). For ACE there was an effect of Group (*F* = 206.04, *P < 0.001, *η*^2^ = 0.812), an effect of Session (*F* = 10.16, *P = 0.002, *η*^2^ = 0.013), and a Group × Session interaction (*F* = 9.41, *P < 0.001, *η*^2^ = 0.037). For DS there was only an effect of Group (*F* = 6.67, *P < 0.001, *η*^2^ = 0.160), but no effect of Session or a Group × Session interaction. *Post hoc* analysis was significant for ACE with respect to session for the Alzheimer’s disease group (*F* = 5.82, *P < 0.001), and for all groups comparisons (*P < 0.001 except EHC versus SCD *P = 0.009), and for DS only for group comparisons between the Alzheimer’s disease group and the other groups (EHC versus Alzheimer’s disease *P < 0.001, SCD versus Alzheimer’s disease *P = 0.003, MCI versus Alzheimer’s disease *P = 0.009).

#### Digital metrics in the prediction of cognitive decline

All metrics at baseline were able to independently predict cognitive decline after 1 year (Supplementary Table 6). The metric that performed the best in predicting a decline in ACE scores after 1 year was memory Imprecision, *t* = 5.5, *P < 0.001, *R*^2^ = 0.411. As a comparison, both ACE itself at baseline and hippocampal volume performed worse than memory Imprecision (ACE: h-test ACE, *z* = 6.34, *P < 0.001, Hippocampal volume: h-test, *z* = 5.74, *P < 0.001).

### Neuroimaging analysis

**Figure 4:**
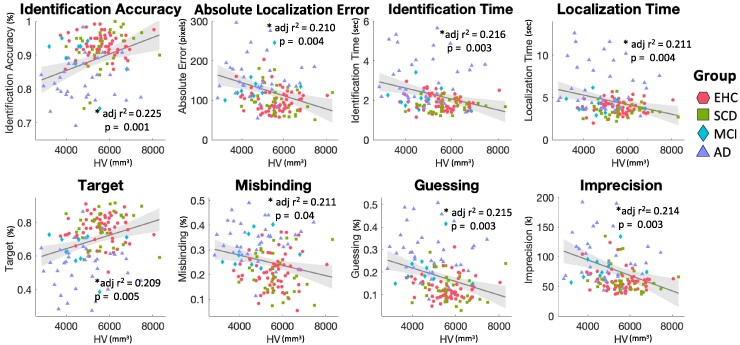
**Correlations between hippocampal volumes and digital metrics.** A generalized linear model was used to study correlations between neuropsychological measures and hippocampal volumes, while correcting for age, gender and education. *r*^2^ represents the overall model fit, the *P*-value refers to the contribution of the metric to the regression. EHC in coral red hexagons, SCD in green squares, MCI in light blue diamonds, Alzheimer’s disease dementia in violet triangles. The regression line has been plotted for the whole dataset, in grey. HV, head-size corrected hippocampal volume, in mm^3^.

All metrics were able to independently predict hippocampal volume in the whole dataset (see Fig. 4). In comparison, age had a smaller effect size compared with Identification accuracy in predicting hippocampal volume in the regression analysis (age: *t* = −2.823, *P = 0.005, Identification accuracy, *t* = 3.251, *P = 0.001, h-test, *z* = −4.3602, *P < 0.001). Moreover, Identification accuracy was the metric that was more tightly correlated with HV compared with the other metrics (h-test, *z* = 3.721, *P < 0.001), and performed as well as ACE (*t* = 3.761, *P < 0.001) in predicting hippocampal volume (h-test, *z* = − 0.190, *P* = 0.8494).

### Linear support vector machine

#### Overall classifier

**Figure 5:**
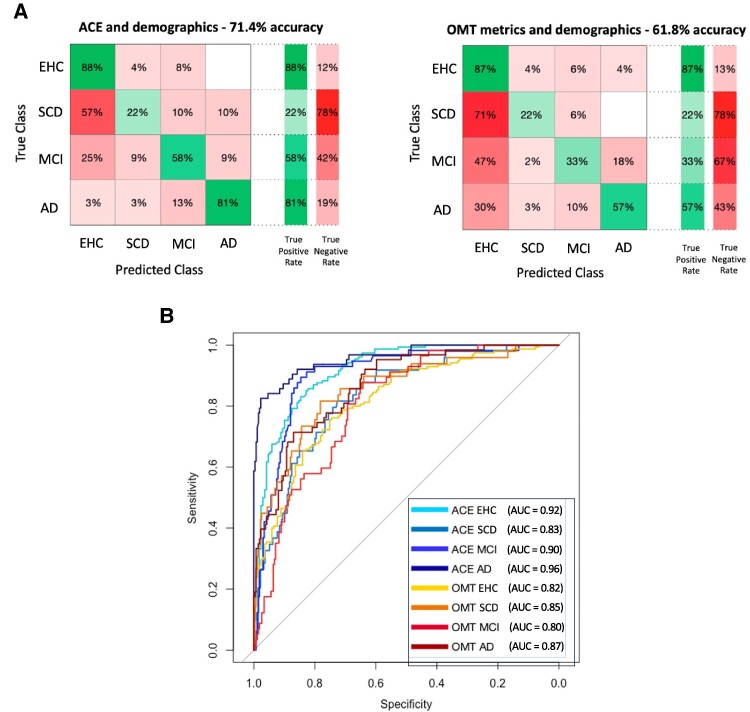
**Linear support vector machine classifier—all groups.** (**A**) Linear support vector machine classifier across all groups. The first model included ACE, age, gender and education, and the second all the eight OMT metrics, age, gender and education. Both models were tested for predicting group classification separately for EHC, SCD, MCI and Alzheimer’s disease dementia. (**B**) Head-to-head comparison of ROC (receiver operating characteristic) curves for ACE and OMT models. Graded blue colours belong to ACE model: light blue = EHC, turquoise = SCD, blue = MCI, dark blue = Alzheimer’s disease dementia. Graded red colour belong to the OMT model: yellow = EHC, orange = SCD, red = MCI, dark red = Alzheimer’s disease dementia.

For the model using the combination of the eight digital metrics (here labelled as OMT), overall accuracy was 61.8%, and the area under the curve (AUC) for predicting group classification was respectively 0.82 for healthy controls, 0.85 for patients with SCD, 0.80 for MCI, and 0.87 for Alzheimer’s disease dementia (Fig. 5A-right and B). In comparison, overall accuracy for the model including ACE was 71.4%, and the AUCs were respectively 0.92 for healthy controls, 0.83 for patients with SCD, 0.90 for MCI and 0.96 for Alzheimer’s disease dementia (Fig. 5A-left and B).

We computed pairwise comparisons using the DeLong method for comparing receiver operating characteristic curves^33^ between the model including OMT and the one including ACE for each group, which were respectively: EHC: *Z* = 4.83, *P < 0.001, SCD: *Z* = −1.34, *P* = 0.179, MCI: *Z* = 3.81, *P < 0.001, Alzheimer’s disease: *Z* = 3.89, *P < 0.001. Therefore, the model using our digital metrics did not perform better than ACE in classifying healthy controls, patients with MCI and Alzheimer’s disease dementia, but was as good as the ACE in patients with SCD (Fig. 5).

#### Pairwise classifier

**Figure 6:**
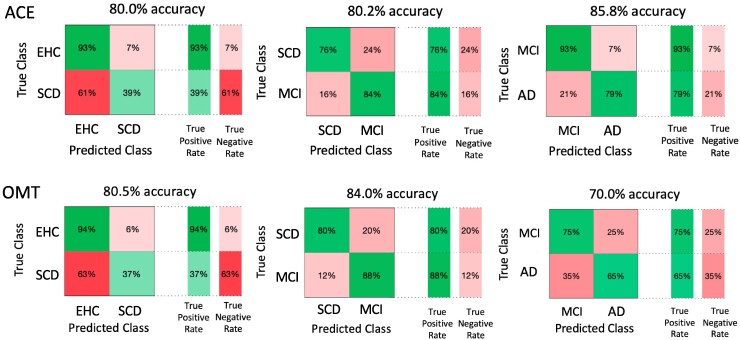
**Linear support vector machine classifier—pairwise.** The first model (ACE) includes ACE, age, gender and education, while the second (OMT) includes all eight OMT metrics, age, gender and education.

Despite these multigroup classifiers being useful for blinded automatic diagnostic classifications, in a clinical setting we are less likely to be presented with a scenario when we have four different diagnoses that could fit the subject’s clinical profile. We therefore performed specific pairwise group comparisons where our metrics could potentially be clinically useful. As shown in Fig. 6, the model including ACE and the one using OMT metrics perform similarly in the differential diagnosis between healthy elderly controls and patients with SCD (AUC ACE = 0.86, AUC OMT = 0.82, *Z* = 1.33, *P* = 0.182) and between SCD and MCI (AUC ACE = 0.91, AUC OMT = 0.92, *Z* = −0.757, *P* = 0.449). However, when examining patients with MCI and Alzheimer’s disease dementia, whilst the ACE performs extremely well, OMT metrics are not as good (AUC ACE = 0.91, AUC OMT = 0.75, *Z* = 2.84, *P = 0.004).

## Discussion

In this study we have shown that digital visual working memory metrics extracted by the ‘What was Where?’ OMT are useful to detect early signs of cognitive impairment in a large cohort of subjects in the Alzheimer’s disease continuum. They are also able to detect disease progression cross-sectionally, between MCI and Alzheimer’s disease dementia, but also longitudinally after 1 year. Identification accuracy seems to be the metric that best reflects the degree of underlying hippocampal atrophy, while Absolute Localization Error and memory Imprecision are useful to predict cognitive decline at standard tests of cognition over one year. Using a linear support vector machine classifier, we found that these metrics seem to perform equally as good as standard tests of cognition, such as the ACE in discriminating between healthy controls, patients with SCD and MCI, while they are not as accurate as ACE in later stages of the disease, such as in the comparison between MCI and Alzheimer’s disease dementia.

All digital metrics were able to discriminate between healthy controls and Alzheimer’s disease dementia patients, as well as between SCD and Alzheimer’s disease dementia (Table 2, Fig. 2). However, no metric was able to distinguish between healthy controls and patients with SCD. This finding is consistent with data that show that the majority of people with a diagnosis of SCD will not subsequently develop Alzheimer’s disease dementia.^18^ Another important finding is that Absolute Localization Error, Target Detection, Misbinding and Guessing were all able to discriminate between patients with SCD and MCI. This might have potentially useful implications, as in clinical practice it can sometimes be difficult to distinguish between these groups on standard tests of cognition.

Both Identification accuracy and memory Imprecision did not differ significantly between healthy controls and patients with SCD, nor between SCD and MCI. However, there was a significant difference between healthy controls and the MCI group. In the comparison between MCI and Alzheimer’s disease dementia, all digital metrics, except Absolute Localization Error, were significantly different. Absolute Localization Error seems to be sensitive to early stages of disease, as it was useful in discriminating between healthy controls and MCI, as well as between SCD and MCI. But beyond the MCI stage, it did not deteriorate significantly further in patients with Alzheimer’s disease dementia. Overall, Absolute Localization Error might be viewed as an ‘*early*’ digital marker because it seems to change more prominently in the early phases of cognitive impairment. Interestingly, it was the only marker that was able to highlight disease progression in asymptomatic mutation carriers in the familial Alzheimer’s disease group studied by Pavisic *et al.*,^5^ while in the same report Identification accuracy only declined in symptomatic patients. This further supports the idea that Absolute Localization Error might be considered as an early marker.

On the other hand, Identification time and Localization time might be viewed as ‘late’ markers, as in our sample they became significantly different only when patients reached the Alzheimer’s disease dementia stage. This is consistent with known effects of slowing of responses, e.g. slower reaction times, in patients with Alzheimer’s disease dementia compared with age-matched elderly controls^34^ and patients with MCI.^35,36^ Evidence of slower reaction times in patients with MCI compared with age-matched controls has been more mixed, and whilst a recent meta-analysis reported slower reaction times in this group,^37^ the results from our study do not support this finding. However, the investigations included in the meta-analysis covered only two diagnostic groups, while here four groups were compared and the analyses were corrected for multiple comparisons, with a lower risk of bias but also possibly a reduced statistical significance in head-to-head differences.

Mixture model metrics, including Target Detection, Guessing and Misbinding, were able not only to distinguish between a healthy status and MCI, but also to discriminate between patients with SCD and MCI and MCI and Alzheimer’s disease dementia, so they were useful metrics to provide an accurate stratification of patients and could be considered as both ‘early’ and ‘late’ markers.

Across groups, set size effects had a major impact on memory recall, showing an effect of large magnitude in all metrics, except for memory Imprecision, where it was of medium magnitude (Supplementary Tables 3 and 4 and Figs 1 and 2). This highlights how consuming STM capacity resources by adding items to remember results in lower performance, not only by reducing the precision with which the items are stored, but also causing slower responses, higher spatial localization error, lower percentages of correctly identified items and targets detected, and higher rates of random guessing and misbinding. Apart from Misbinding, all other metrics also showed an effect of delay, which however was of small to medium magnitude. This points towards the fact that remembering an increasing number of items has a much more detrimental impact on memory recall compared with time-dependent memory degradation. A significant effect of set size on memory performance is in line with what previously reported with the same paradigm in healthy controls and patients with sporadic Alzheimer’s disease and Parkinson’s disease.^9,10^ However, the latter studies either did not have sufficient data to examine the effect of delay,^10^ or investigated only a limited subset of these metrics and included a large number of healthy controls but a very small number of patients with either Alzheimer’s disease or Parkinson’s disease (*n* = 19 each).^9^ This study replicates these findings in a larger clinical cohort of patients with memory impairment.

Measuring changes in our cognitive metrics after one year revealed that they show different profiles: some tend to remain very stable over time, while others follow a similar trajectory as the decline in performance on the ACE (Fig. 3 and Supplementary Fig. 3). The metrics that best tracked individual changes in cognition over time were respectively Absolute Localization Error, but more so, memory Imprecision. Indeed, memory Imprecision performed very well in the longitudinal tracking of cognitive decline, so well that its baseline values outperformed baseline levels of ACE and HV (Supplementary Table 6). One of the most interesting results was that despite a decline in cognition, indexed by a reduction in ACE scores, mixture model metrics (Target detection, Misbinding and Guessing) did not change over time. Why is that so? To answer this question, we need to bear in mind how these metrics are calculated. They do not reflect absolute values of errors, but they calculate the probability of a response belonging to correct target identification, misbinding and guessing, which all add up to 1, as they are relative to each other. Therefore, the most likely explanation is that the proportion of mistakes made, ‘relative to each other’, remains the same across time.

Previous evidence has pointed towards Misbinding as being the most important marker of hippocampal integrity across different clinical populations.^4,11^ While we could replicate that misbinding rates are indeed associated with hippocampal atrophy, this was not the best predictor among the digital metrics used for this experiment. Here, Identification accuracy stood out as the metric that most strongly correlated with hippocampal volume, performing equally as well as validated standard neuropsychological tests such as the ACE (Fig. 4). It is also worth noting that these results were corrected for common confounding factors that could impact performance as well as hippocampal integrity, such as age, gender and levels of education. More importantly, Identification accuracy explained more variance compared to ageing in predicting hippocampal volume in this dataset, which is also encouraging.

Regarding head-to-head comparisons between digital metrics and standard tests of cognition, while ACE was overall superior to OMT metrics and better in the discrimination between MCI and Alzheimer’s disease dementia, digital metrics seem to perform similarly to traditional pen-and-paper tests when comparing healthy controls to SCD patients, or SCD to MCI (Figs 5 and 6). This has potentially useful clinical implications as this test can now be administered fully online and remotely, without requiring dedicated, time-consuming face-to-face appointments, as shown by a subsequent web-based version of this task in a cohort of healthy controls and early Alzheimer’s disease dementia patients.^21^ Further studies are needed to compare the performance of a web-based version of this task in patients with SCD and MCI to standard neuropsychological tests.

There are some limitations to this study. Only 26/63 patients with Alzheimers’s disease dementia had biological confirmation of Alzheimer’s disease biomarkers, which confirmed presence of amyloid and tau positivity. While this sample is representative of patients seen in a memory clinic, subsequent studies should aim to validate these findings in a fully biomarker-validated cohort. Moreover, the longitudinal dataset had a limited number of participants, due to the study being interrupted in response to COVID-19 restrictions. Another limitation of the study was that the cohort of patients with subjective cognitive decline was younger than the other cohorts. Patients with subjective cognitive decline presenting to memory clinics are on average younger than patients with organic causes of cognitive impairment,^18^ which is an intrinsic limitation of studies involving this cohort. We tried to control for this confounder by using age as covariate throughout the analyses. Additionally, participants enrolled in the longitudinal dataset were all non-converters, i.e. none of them had their diagnosis changed at their follow-up visit after 1 year. A much larger longitudinal dataset is needed to replicate these findings and to study which metric might be a better marker of clinical conversion from early stages (e.g. SCD and MCI) to Alzheimer’s disease dementia.

## Supplementary Material

fcaf024_Supplementary_Data

## Data Availability

De-identified data supporting this study may be shared based on reasonable written requests to the corresponding author. Access to de-identified data will require a Data Access Agreement and IRB clearance, which will be considered by the institutions who provided the data for this research.
