# Supplementary material for: Performance and validation of a digital memory test across the Alzheimer’s disease continuum
Source: Brain Commun. 2025 Jan 17;7(1):fcaf024. doi: 10.1093/braincomms/fcaf024 (PMC11780857; doi:10.1093/braincomms/fcaf024)
Supplement: fcaf024_Supplementary_Data [file fcaf024_supplementary_data.docx]

**SUPPLEMENTARY MATERIALS**

|  | **EHC** | **SCD** | **MCI** | **AD** | **p-value all** | **p-value EHC/SCD** | **p-value SCD/MCI** | **p-value MCI/AD** |
| --- | --- | --- | --- | --- | --- | --- | --- | --- |
|  |  |  |  |  |  |  |  |  |
| **Age** | 66.6 (7.5) | 59.9 (8.6) | 69.3 (11.5) | 69.8 (11.2) | *0.040 | 0.165 | 0.065 | 1 |
| **Gender (M/F)** | 6/15 | 8/7 | 8/4 | 5/7 | 0.175 | 0.714 | 1 | 0.877 |
| **Education** | 15.8 (3.6) | 15.9 (5.2) | 14.0 (4.7) | 15.5 (6.2) | 0.448 | 1 | 1 | 1 |
|  |  |  |  |  |  |  |  |  |
| **ACE** | 97.4 (2.0) | 94.3 (3.5) | 86.7 (7.7) | 71.7 (5.2) | *<0.001 | 0.052 | *<0.001 | *<0.001 |
| **DS** | 17.7 (2.9) | 18.2 (3.8) | 17.2 (4.8) | 13.8 (2.2) | *0.007 | 1 | 1 | 0.079 |

**Supplementary Table 1 | Demographics and tests – longitudinal subset**

P-values are derived by the between-group ANOVA (p-value all) with subsequent Holm post-hoc comparisons (p-value EHC/SCD, p-value SCD/MCI, p-value MCI/AD).

|  | | **EHC** | **SCD** | **MCI** | **AD** | **p-value all** | **p-value EHC/SCD** | **p-value SCD/MCI** | **p-value MCI/AD** |
| --- | --- | --- | --- | --- | --- | --- | --- | --- | --- |
|  | **Age** | 66.6 (7.5) | 57.7 (7.2) | 65.8 (11.3) | 69.2 (9.7) | *<0.001 | *<0.001 | *0.048 | 0.546 |
|  | **Gender (M/F)** | 24/37 | 14/17 | 4/5 | 22/15 | 0.285 | 0.592 | 0.969 | 0.415 |
|  | **Education** | 16.2 (3.8) | 16.0 (4.5) | 16.6 (4.4) | 14.1 (3.6) | *0.046 | 1 | 1 | 0.411 |
|  | **ACE** | 97.2 (2.5) | 93.9 (5.7) | 89.6 (3.9) | 70.5 (19.3) | *<0.001 | 0.335 | 0.335 | *<0.001 |

**Supplementary Table 2 | Demographics and tests – neuroimaging sample**

P-values are derived by the between-group ANOVA (p-value all) with subsequent Holm post-hoc comparisons (p-value EHC/SCD, p-value SCD/MCI, p-value MCI/AD).

**EFFECTS OF SET SIZE AND DELAY**

|  | **Set size** | **Delay** | **Set size x Delay** |
| --- | --- | --- | --- |
|  |  |  |  |
| **Identification Accuracy** | F = 1298.7, ***p < 0.001**  η2 = 0.624 | F = 168.2, ***p < 0.001**  η2 = 0.043 | F = 20.7, ***p < 0.001**  η2 = 0.006 |
| **Absolute Localization Error** | F = 1625.5, ***p < 0.001**  η2 = 0.726 | F = 109.5, ***p < 0.001**  η2 = 0.019 | F = 35.0, ***p < 0.001**  η2 = 0.005 |
| **Identification Time** | F = 493.6, ***p < 0.001**  η2 = 0.466 | F = 122.0, ***p < 0.001**  η2 = 0.037 | F = 4.1, ***p = 0.043**  η2 = 0.001 |
| **Localization Time** | F = 423.2, ***p < 0.001**  η2 = 0.416 | F = 161.8, ***p < 0.001**  η2 = 0.046 | F = 1.8, p = 0.186 |
| **Target detection** | F = 3088.8, ***p < 0.001**  η2 = 0.845 | F = 34.1, ***p < 0.001**  η2 = 0.004 | F = 0.008, p = 0.927 |
| **Misbinding** | N/A | F = 0.3, p = 0.577 | N/A |
| **Guessing** | F = 145.0, ***p < 0.001**  η2 = 0.211 | F = 96.6, ***p < 0.001**  η2 = 0.045 | F = 1.3, p = 0.249 |
| **Imprecision** | F = 49.6, ***p < 0.001**  η2 = 0.085 | F = 78.8, ***p < 0.001**  η2 = 0.043 | F = 5.0, ***p = 0.001**  η2 = 0.002 |

^1^Statistically significant values are represented in bold.

**Supplementary Table 3 | Cross-sectional analysis of Set size and Delay effects**

A 2 (Set size: 1 item, 3 items) x 2 (Delay: 1 second, 4 seconds) ANOVA was performed for each of the digital working memory metrics. Set size (1 vs 3 items), Delay (1 vs 4 seconds), N/A = not applicable.

|  | **Metric** | **Set size** | **Delay** | **Set size x Delay** |
| --- | --- | --- | --- | --- |
| **Group EHC** | **Identification Accuracy** | F = 664.2, ***p < 0.001**  η2 = 0.605 | F = 89.5, ***p < 0.001**  η2 = 0.057 | F = 23.9, ***p < 0.001**  η2 = 0.013 |
|  | **Absolute**  **Localization Error** | F = 827.3, ***p < 0.001**  η2 = 0.732 | F = 64.2, ***p < 0.001**  η2 = 0.022 | F = 28.5, ***p < 0.001**  η2 = 0.009 |
|  | **Identification Time** | F = 572.5, ***p < 0.001**  η2 = 0.641 | F = 118.3, ***p < 0.001**  η2 = 0.051 | F = 6.0, ***p = 0.016**  η2 = 0.002 |
|  | **Localization Time** | F = 580.2, ***p < 0.001**  η2 = 0.634 | F = 176.7, ***p < 0.001**  η2 = 0.070 | F = 2.4, p = 0.125 |
|  | **Target detection** | F = 1389.5, ***p < 0.001**  η2 = 0.838 | F = 27.6, ***p < 0.001**  η2 = 0.006 | F = 3.828, p = 0.052 |
|  | **Misbinding** | N/A | F = 6.107, ***p = 0.015** η2 = 0.038 | N/A |
|  | **Guessing** | F = 229.0, ***p < 0.001**  η2 = 0.444 | F = 50.1, ***p < 0.001**  η2 = 0.036 | F = 0.7, p = 0.407 |
|  | **Imprecision** | F = 181.6, ***p < 0.001**  η2 = 0.370 | F = 49.7, ***p < 0.001**  η2 = 0.042 | F = 2.3, p = 0.131 |
|  |  |  |  |  |
| **Group SCD** | **Identification Accuracy** | F = 152.5, ***p < 0.001**  η2 = 0.590 | F = 20.2, ***p < 0.001**  η2 = 0.041 | F = 4.7, ***p = 0.035**  η2 = 0.008 |
|  | **Absolute Localization Error** | F = 212.8, ***p < 0.001**  η2 = 0.732 | F = 9.3, ***p = 0.004**  η2 = 0.010 | F = 4.5, ***p = 0.038**  η2 = 0.004 |
|  | **Identification Time** | F = 125.8, ***p < 0.001**  η2 = 0.592 | F = 33.8, ***p < 0.001**  η2 = 0.052 | F = 14.7, ***p < 0.014**  η2 = 0.014 |
|  | **Localization Time** | F = 107.9 ***p < 0.001**  η2 = 0.554 | F = 46.6, ***p < 0.001**  η2 = 0.071 | F = 14.5, ***p < 0.001**  η2 = 0.013 |
|  | **Target detection** | F = 328.4, ***p < 0.001**  η2 = 0.808 | F = 2.2, p = 0.148 | F = 0.000, p = 0.980 |
|  | **Misbinding** | N/A | F = 0.1, p = 0.703 | N/A |
|  | **Guessing** | F = 58.9, ***p < 0.001**  η2 = 0.420 | F = 6.9, ***p = 0.011**  η2 = 0.020 | F = 1.1, p = 0.308 |
|  | **Imprecision** | F = 38.5, ***p < 0.001**  η2 = 0.334 | F = 6.0, ***p = 0.018**  η2 = 0.019 | F = 0.6, p = 0.450 |
|  |  |  |  |  |
| **Group MCI** | **Identification Accuracy** | F = 409.3, ***p < 0.001**  η2 = 0.730 | F = 27.1, ***p < 0.001**  η2 = 0.030 | F = 1.5, p = 0.218 |
|  | **Absolute Localization Error** | F = 589.0, ***p < 0.001**  η2 = 0.730 | F = 10.6, ***p = 0.002**  η2 = 0.009 | F = 4.2, ***p = 0.046**  η2 = 0.003 |
|  | **Identification Time** | F = 114.5, ***p < 0.001**  η2 = 0.546 | F = 15.0, ***p < 0.001**  η2 = 0.019 | F = 1.5, p = 0.222 |
|  | **Localization Time** | F = 108.0, ***p < 0.001**  η2 = 0.524 | F = 14.3, ***p < 0.001**  η2 = 0.022 | F = 0.8, p = 0.385 |
|  | **Target detection** | F = 948.7, ***p < 0.001**  η2 = 0.889 | F = 1.0, p = 0.309 | F = 1.784, p = 0.187 |
|  | **Misbinding** | N/A | F = 2.8, p = 0.099 | N/A |
|  | **Guessing** | F = 27.0, ***p < 0.001**  η2 = 0.201 | F = 20.5, ***p < 0.001**  η2 = 0.070 | F = 0.3, p = 0.560 |
|  | **Imprecision** | F = 19.4, ***p < 0.001**  η2 = 0.142 | F = 17.9, ***p < 0.001**  η2 = 0.069 | F = 0.2, p = 0.618 |
|  |  |  |  |  |
| **Group AD** | **Identification Accuracy** | F =322.2, ***p < 0.001**  η2 = 0.661 | F = 32.8, ***p < 0.001**  η2 = 0.039 | F = 0.4, p = 0.534 |
|  | **Absolute Localization Error** | F = 288.6, ***p < 0.001**  η2 = 0.683 | F = 33.4, ***p < 0.001**  η2 = 0.036 | F = 3.0, p = 0.081 |
|  | **Identification Time** | F = 27.1, ***p < 0.001**  η2 = 0.211 | F = 12.0, ***p < 0.001**  η2 = 0.029 | F = 0.7, p = 0.413 |
|  | **Localization Time** | F = 21.5, ***p < 0.001**  η2 = 0.164 | F = 16.9, ***p < 0.001**  η2 = 0.033 | F = 0.1, p = 0.748 |
|  | **Target detection** | F = 816.3, ***p < 0.001**  η2 = 0.871 | F = 8.1, ***p = 0.006**  η2 = 0.004 | F = 2.798, p = 0.099 |
|  | **Misbinding** | N/A | F = 0.9, p = 0.341 | N/A |
|  | **Guessing** | F = 0.6, *p = 0.443  η2 = 0.006 | F = 26.6, ***p < 0.001**  η2 = 0.069 | F = 0.9, p = 0.342 |
|  | **Imprecision** | F = 2.0, p = 0.001  η2 = 0.020 | F = 22.1, ***p < 0.001**  η2 = 0.061 | F = 4.3, ***p = 0.042**  η2 = 0.009 |

^1^Statistically significant values are represented in bold.

**Supplementary Table 4 | Group-by-group Set size and Delay effects**

A 2 (Set size: 1 item, 3 items) x 2 (Delay: 1 second, 4 seconds) ANOVA was performed for each of the digital working memory metrics within each group. EHC = elderly healthy controls, SCD = subjective cognitive decline, MCI = mild cognitive impairment, AD = Alzheimer’s Disease dementia.

**
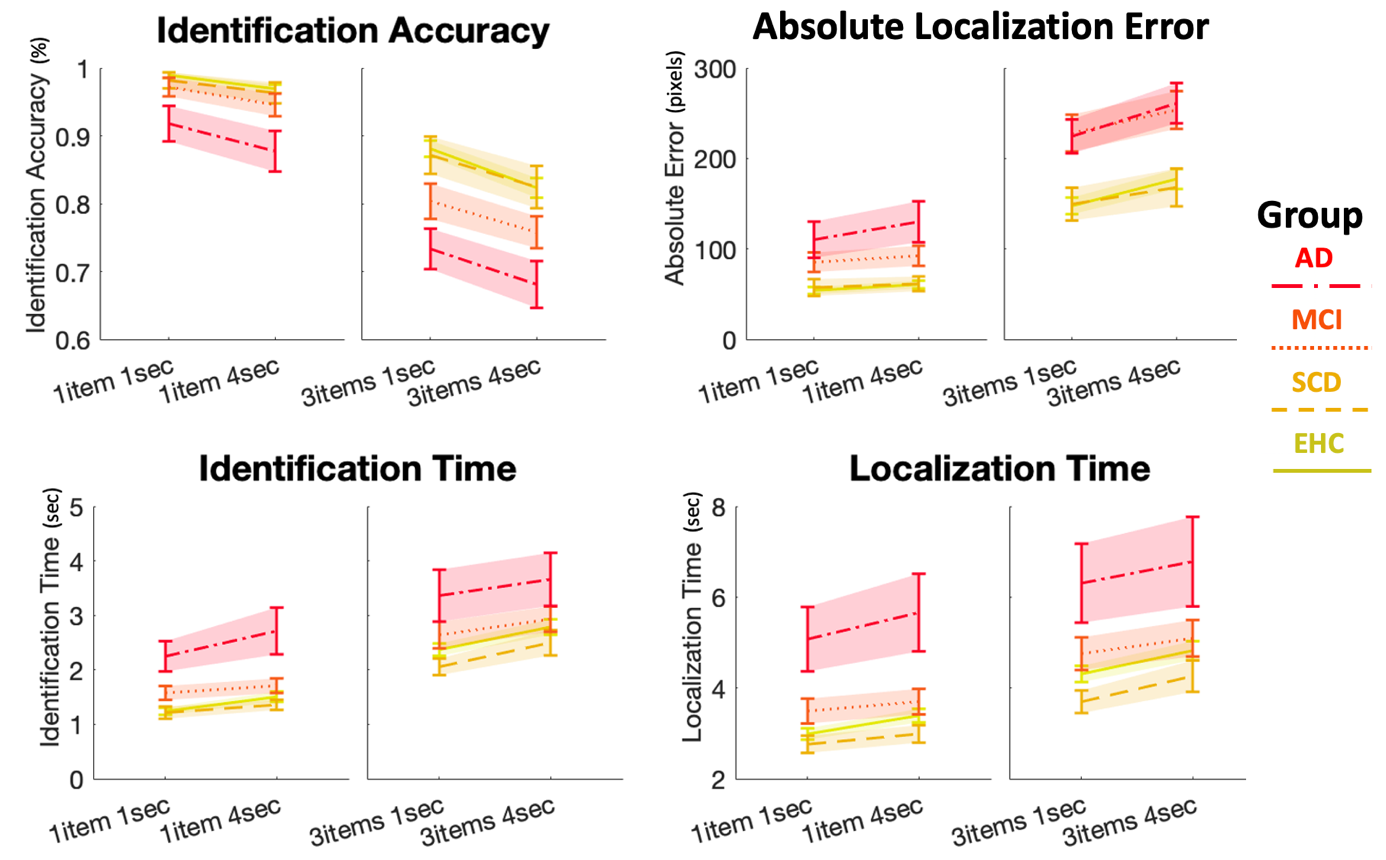
**

**Supplementary Figure 1 | Transdiagnostic analysis: Effects of Set Size and Delay - basic metrics**

A 2 (Set size: 1 item, 3 items) x 2 (Delay: 1 second, 4 seconds) ANOVA was performed for each of the digital working memory metrics, with Holm post-hoc correction across groups. Statistical values are provided in Supplementary Table 3 and Supplementary Table 4. EHC = yellow solid line, SCD = light orange dashed line, MCI = dark orange dotted line, AD = red dash-dotted line.


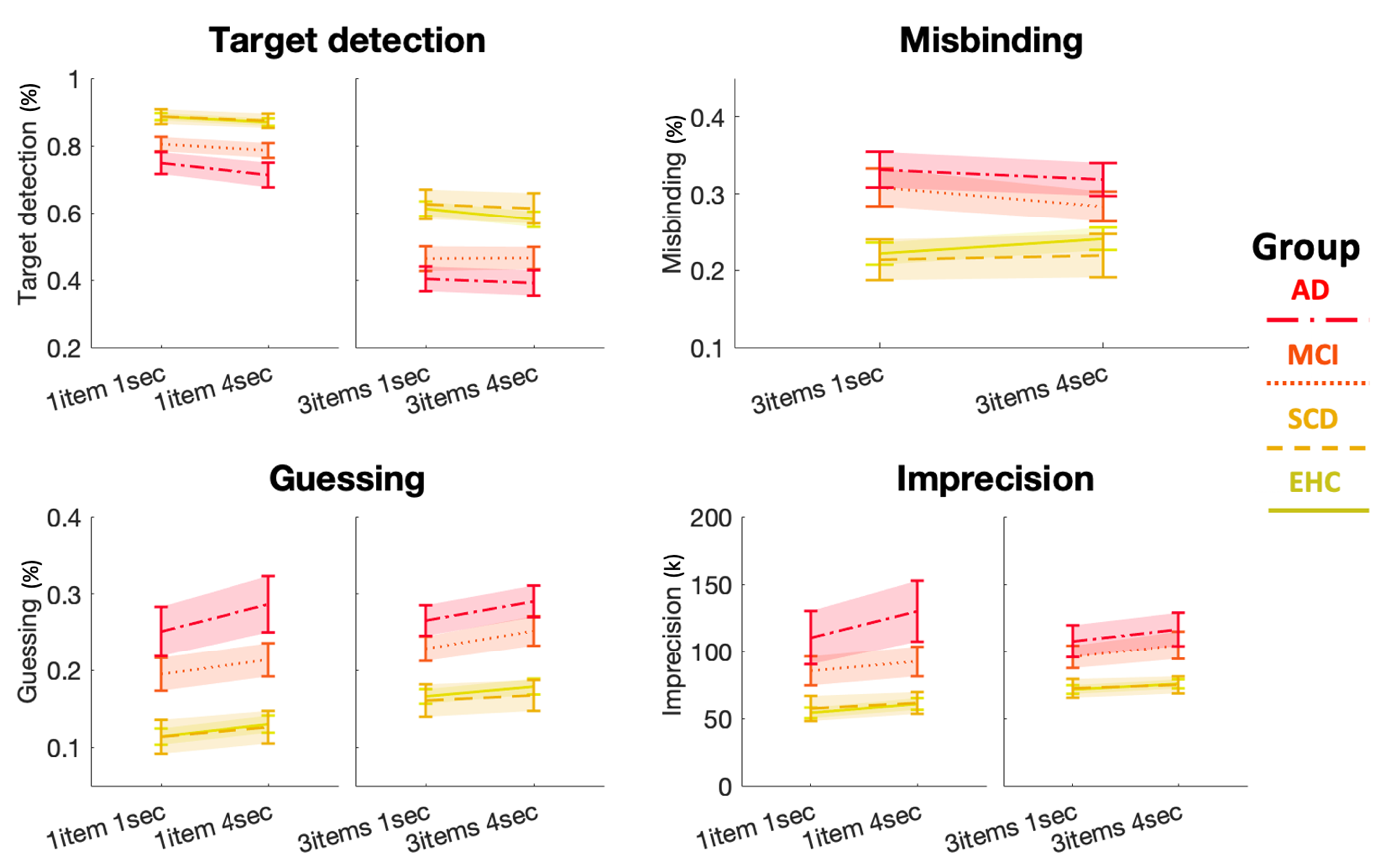


**Supplementary Figure 2 | Transdiagnostic analysis: Effects of Set Size and Delay - mixture model metrics**

A 2 (Set size: 1 item, 3 items) x 2 (Delay: 1 second, 4 seconds) ANOVA was performed for each of the digital working memory metrics, with Holm post-hoc correction across groups. Statistical values are provided in Supplementary Table 3 and Supplementary Table 4. EHC = yellow solid line, SCD = light orange dashed line, MCI = dark orange dotted line, AD = red dash-dotted line.

|  | **Group** | **Session** | **Group x Session** |
| --- | --- | --- | --- |
|  |  |  |  |
| **Identification Accuracy** | F = 46.6, ***p < 0.001**  η2 = 0.551 | n.s | n.s |
| **Absolute Localization Error** | F = 64.9, ***p < 0.001**  η2 = 0.614 | n.s | F = 3.2, ***p = 0.025**  η2 = 0.031 |
| **Identification Time** | F = 35.0, ***p < 0.001**  η2 = 0.472 | n.s | n.s |
| **Localization Time** | F = 20.3, ***p < 0.001**  η2 = 0.333 | F = 4.3, ***p = 0.039**  η2 = 0.024 | n.s |
| **Target detection** | F = 72.7, ***p < 0.001**  η2 = 0.667 | n.s | n.s |
| **Guessing** | F = 67.6, ***p < 0.001**  η2 = 0.640 | n.s | n.s |
| **Misbinding** | F = 28.9, ***p < 0.001**  η2 = 0.447 | n.s | n.s |
| **Imprecision** | F = 45.9, ***p < 0.001**  η2 = 0.507 | n.s | F = 4.8, ***p = 0.004**  η2 = 0.053 |

**Supplementary Table 5 | Longitudinal dataset - Group and Session effects**

A 4 (Group) x 2 (Session) ANCOVA, with age, gender and education as covariates, with subsequent Holm post-hoc correction was used to test differences across groups and sessions. Session represents baseline session (Time 0) and follow-up session after 1 year. n.s. = not significant.


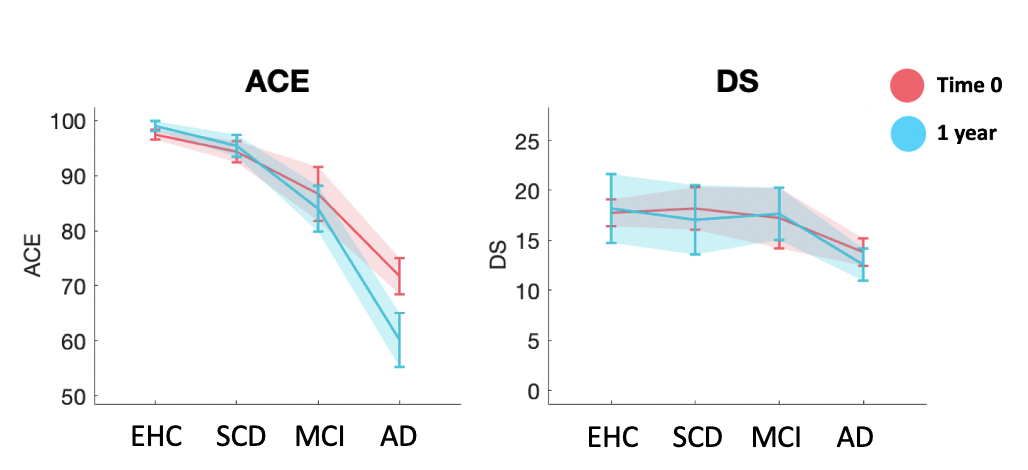


**Supplementary Figure 3 | Longitudinal analysis of standard neuropsychological tests**

A 4 (Group) x 2 (Session) ANCOVA, with age, gender and education as covariates, with subsequent Holm post-hoc correction was used to test differences across groups and sessions. ACE: main effect of Group (F = 206.04, *p < 0.001, η2 = 0.812), Session (F = 10.16, *p = 0.002, η2 = 0.013), and a Group x Session interaction (F = 9.41, *p < 0.001, η2 = 0.037). DS: main effect of Group (F = 6.67, *p < 0.001, η2 = 0.160). Post-hoc analysis for ACE was significant for session in the AD group (F = 5.82, *p < 0.001), and for all groups comparisons (*p <0.001 except EHC vs SCD *p = 0.009), and for DS only for group comparisons between the AD group and the other groups (EHC vs AD *p <0.001, SCD vs AD *p = 0.003, MCI vs AD *p = 0.009). Baseline session (Time 0) in coral red, follow-up session after 1 year (1 year) in light blue. ACE = Addenbrooke’s cognitive examination. DS = Digit Span. EHC = elderly healthy controls, SCD = subjective cognitive decline, MCI = mild cognitive impairment, AD = Alzheimer’s Disease dementia.

|  | **Decline in ACE scores** |
| --- | --- |
|  |  |
| **Identification accuracy** | F (1,45), t = -5.0, *p < 0.001, R^2^ = 0.3537 |
| **Absolute Localization Error** | F (1,45), t = 5.3, *p < 0.001, R^2^ = 0.3537 |
| **Identification time** | F (1,45), t = 4.5, *p = 0.002, R^2^ = 0.3143 |
| **Localization time** | F (1,45), t = 4.6, *p =0.001, R^2^ = 0.3239 |
| **Target detection** | F (1,45), t = -5.2, *p < 0.001, R^2^ = 0.3902 |
| **Misbinding** | F (1,45), t = 4.0, *p < 0.001, R^2^ = 0.2706 |
| **Guessing** | F (1,45), t = 4.6, *p = 0.005, R^2^ = 0.3333 |
| **Imprecision** | F (1,45), t = 5.5, *p < 0.001, R^2^ = 0.4107 |
|  |  |

**Supplementary Table 6 | Longitudinal dataset – prediction of cognitive decline**

Linear regression was used to test the ability of each metric to predict cognitive decline at the Addenbrooke’s cognitive examination (ACE) at 1 year.
